# Synergic prodegradative activity of Bicalutamide and trehalose on the mutant androgen receptor responsible for spinal and bulbar muscular atrophy

**DOI:** 10.1093/hmg/ddu419

**Published:** 2014-08-13

**Authors:** Elisa Giorgetti, Paola Rusmini, Valeria Crippa, Riccardo Cristofani, Alessandra Boncoraglio, Maria E. Cicardi, Mariarita Galbiati, Angelo Poletti

**Affiliations:** 1 Sezione di Biomedicina ed Endocrinologia, Dipartimento di Scienze Farmacologiche e Biomolecolari (DiSFeB), Centro di Eccellenza sulle Malattie Neurodegenerative, Università degli Studi di Milano, Milano 20133, Italy; 2 Centro InterUniversitario sulle Malattie Neurodegenerative, Università degli Studi di Firenze, Genova e Roma Tor Vergata, Milano 20133, Italy; 3 Department of Pathology, University of Michigan, Ann Arbor, MI 48109, USA and; 4 Department of Cell Biology, University Medical Center of Groningen, RB 9700 Groningen, The Netherlands

## Abstract

Spinal and bulbar muscular atrophy (SBMA) is an X-linked motoneuron disease due to a CAG triplet-repeat expansion in the androgen receptor (AR) gene, which is translated into an elongated polyglutamine (polyQ) tract in AR protein (ARpolyQ). ARpolyQ toxicity is activated by the AR ligand testosterone (or dihydrotestosterone), and the polyQ triggers ARpolyQ misfolding and aggregation in spinal cord motoneurons and muscle cells. In motoneurons, testosterone triggers nuclear toxicity by inducing AR nuclear translocation. Thus, (i) prevention of ARpolyQ nuclear localization, combined with (ii) an increased ARpolyQ cytoplasmic clearance, should reduce its detrimental activity. Using the antiandrogen Bicalutamide (Casodex^®^), which slows down AR activation and nuclear translocation, and the disaccharide trehalose, an autophagy activator, we found that, in motoneurons, the two compounds together reduced ARpolyQ insoluble forms with higher efficiency than that obtained with single treatments. The ARpolyQ clearance was mediated by trehalose-induced autophagy combined with the longer cytoplasmic retention of ARpolyQ bound to Bicalutamide. This allows an increased recognition of misfolded species by the autophagic system prior to their migration into the nucleus. Interestingly, the combinatory use of trehalose and Bicalutamide was also efficient in the removal of insoluble species of AR with a very long polyQ (Q112) tract, which typically aggregates into the cell nuclei. Collectively, these data suggest that the combinatory use of Bicalutamide and trehalose is a novel approach to facilitate ARpolyQ clearance that has to be tested in other cell types target of SBMA (i.e. muscle cells) and *in vivo* in animal models of SBMA.

## INTRODUCTION

Spinal and bulbar muscular atrophy (SBMA) or Kennedy's disease is an inherited X-linked motoneuron disease characterized by lower motoneuron degeneration in anterior horns of the spinal cord and in brainstem (1,2). Dorsal root ganglia neurons are also affected causing sensory disturbances (3). Motoneuron loss results in atrophy of bulbar, facial and limb muscles (4,5). Recent data suggest that muscle atrophy is not only an indirect consequence of denervation induced by motoneuron degeneration, but also depends on direct alterations occurring in muscle cells (6–11). SBMA is linked to an expanded CAG triplet-repeat sequence in the androgen receptor (AR) gene, which is translated into an elongated polyglutamine (polyQ) tract in the N-terminus of the AR protein (ARpolyQ) (4). The polyQ tract ranges from 9 to 37 polyglutamines (Qs) (average 22) in normal individuals and is longer than 38 (up to 70) Qs in SBMA patients (4). Interestingly, eight other dominant neurodegenerative diseases (NDs) are linked to similar alterations, but in totally unrelated proteins, lacking any homology or common functional domain with AR and between them. Thus, a polyQ-associated gain of neurotoxic function(s) conferred to these proteins is causative for these NDs (6); for example, the elongated polyQ tract acquires aberrant conformations, which may misfold the mutant proteins, generating species toxic to cells (motoneuron or muscle in SBMA); these species aggregate and form intracellular inclusions. In SBMA patients, ARpolyQ inclusions are present in nuclei of spinal cord motoneurons and in cytoplasm of dorsal root ganglia sensory neurons (12,13). Inclusions are detectable in skeletal muscle cells, which are also targets of ARpolyQ toxicity. Indeed, antisense oligonucleotides specifically suppressing peripheral, but not central nervous system (CNS), AR gene expression rescued muscle deficits extending lifespan of a male mouse (knock-in) model of SBMA (11); muscle-specific excision of human AR121Q present in a bacterial artificial chromosome (BAC) vectors transgenic mouse model of SBMA resulted in a full rescue from the typical aberrant phenotype, including prevention of weight loss, motor phenotypes, muscle pathology and motoneuropathy. Selective AR121Q excision from muscle in BAC fxAR121 dramatically extended survival, thus confirming the role of ARpolyQ in muscle pathology as a contributing factor in SBMA (10). It must be recalled that SBMA mice obtained using a PrP promoter, in which ARpolyQ (14–16) is highly expressed in the CNS, but not in muscle, are also characterized by a dramatic SBMA phenotype, suggesting that both types of cells are involved in the onset and progression of the disease.

In all cases, SBMA has unique features that confer two advantages to study polyQ toxicity. First, AR structure, functions and mechanism of action are very well known (17), allowing one to discriminate between physiological and pathological ARpolyQ behaviors; secondly, ARpolyQ toxicity strictly depends on androgens (i.e. testosterone); thus, ARpolyQ can be switched from a ‘nontoxic’ to ‘neurotoxic’ status, simply by adding testosterone (17–19). In fact, SBMA occurs only in men, and surgical or chemical (with the gonadotropin-releasing hormone, GnRH agonist Leuprorelin) castration ameliorates the phenotype in SBMA male mice (15,19–21), whereas testosterone induces SBMA symptoms in females (18). Unfortunately, the possible benefit of Leuprorelin in SBMA patients is unclear because of the large symptom variability in humans and the very slow progression rate of SBMA (3,22,23). Dutasteride, an inhibitor of the 5-alpha reductase [an enzyme highly expressed in spinal cord motoneurons (24)], which reduces testosterone conversion to its more potent derivative, dihydrotestosterone, has also been tested in SBMA patients, but again with unclear results, same as in Leuprorelin studies (5,25). A very recent work, performed on three different mice models of SBMA, suggested that the antiandrogen flutamide could partly counteract ARpolyQ toxicity in SBMA (26), suggesting a potential for AR antagonists usage in SBMA. Other studies have demonstrated the potential benefits of selective AR modulators in cell models of SBMA (27).

All these studies are based on the fact that, in the absence of testosterone, ARpolyQ localizes in the cytoplasm where it forms a multi-heteromeric complex with chaperones [heat shock protein (HSP)90, HSP70, etc.]. In this complex, AR would be in a nontoxic ‘immature’ status, with the polyQ tract possibly ‘masked’ through the interaction with other proteins. To activate AR, testosterone induces the release from chaperones allowing the conformational changes required for AR nuclear translocation and function; however, the polyQ tract may reduce the efficiency of this process causing protein misfolding. Misfolded ARpolyQ then exerts most of its toxicity in the nucleus (28–31). Thus, prevention of ARpolyQ misfolding and nuclear translocation may counteract its neurotoxicity (see below). Misfolded ARpolyQ species may directly alter the degradative pathways in affected cells. In fact, while cytoplasmic soluble ARpolyQ (testosterone-untreated) impairs the ubiquitin–proteasome system (UPS), testosterone-induced ARpolyQ aggregation correlates with normal UPS activity, induction of autophagy (6–8,32–36) and also with a blockage of the autophagic flux (37,38). Thus, the aggregation process, by physically sequestering neurotoxic misfolded species into a defined subcellular compartment (‘the aggregates’), may be initially protective to cells (17,37,39–41); but at later stages, they may affect essential neuronal processes and become neurotoxic. In any case, ARpolyQ aggregates represent a valuable marker to monitor the formation/clearance of toxic misfolded species in affected cells. In addition, if not removed by cytoplasmic autophagy, a large amount of misfolded ARpolyQ goes into the nucleus where it can exert neurotoxicity. In fact, Montie *et al.* (28,29) already demonstrated that cytoplasmic ARpolyQ retention correlates with increased neuronal survival in cells and improved motor functions in SBMA mice.

Others (27) and we (37,38) found that testosterone-induced ARpolyQ misfolding and aggregation can be prevented/counteracted by some antiandrogens, like Bicalutamide, an FDA-approved antiandrogen, commercialized as Casodex^®^ (Cas). Bicalutamide reduces the rate of ARpolyQ nuclear translocation, allowing its more efficient cytoplasmic degradation via autophagy (27,37,38,41).

The disaccharide trehalose enhances ARpolyQ (and other misfolded proteins) degradation (37,42–47), extending life span of different mouse models of NDs (42,43,45,48).

Since cytoplasmic ARpolyQ retention is beneficial in SBMA models because it increases autophagic clearance of the misfolded ARpolyQ (28,29,37,41,49); in this study, we tested a combination of Bicalutamide (which retains ARpolyQ into the cytoplasm, by slowing down its nuclear translocation rate) (27,37,38,41) and trehalose (which stimulates HSPB8 expression and enhances autophagy). Since the molecular mechanisms that mediate ARpolyQ neurotoxicity in motoneurons have been extensively studied, while the alterations responsible for the recently described muscle toxicity of ARpolyQ are still unknown, we decided to focus our attention on the ARpolyQ alteration which are induced in motoneuronal cells, rather than in muscle cells. Our data demonstrate that the combinatory use of Bicalutamide and trehalose has a potent synergic activity against ARpolyQ accumulation in neuronal models of SBMA: it efficiently decreases the number of aggregates and accelerates ARpolyQ clearance; thus, this combined treatment could provide a novel valuable approach to counteract ARpolyQ toxicity in SBMA.

## RESULTS

**Figure 1.**
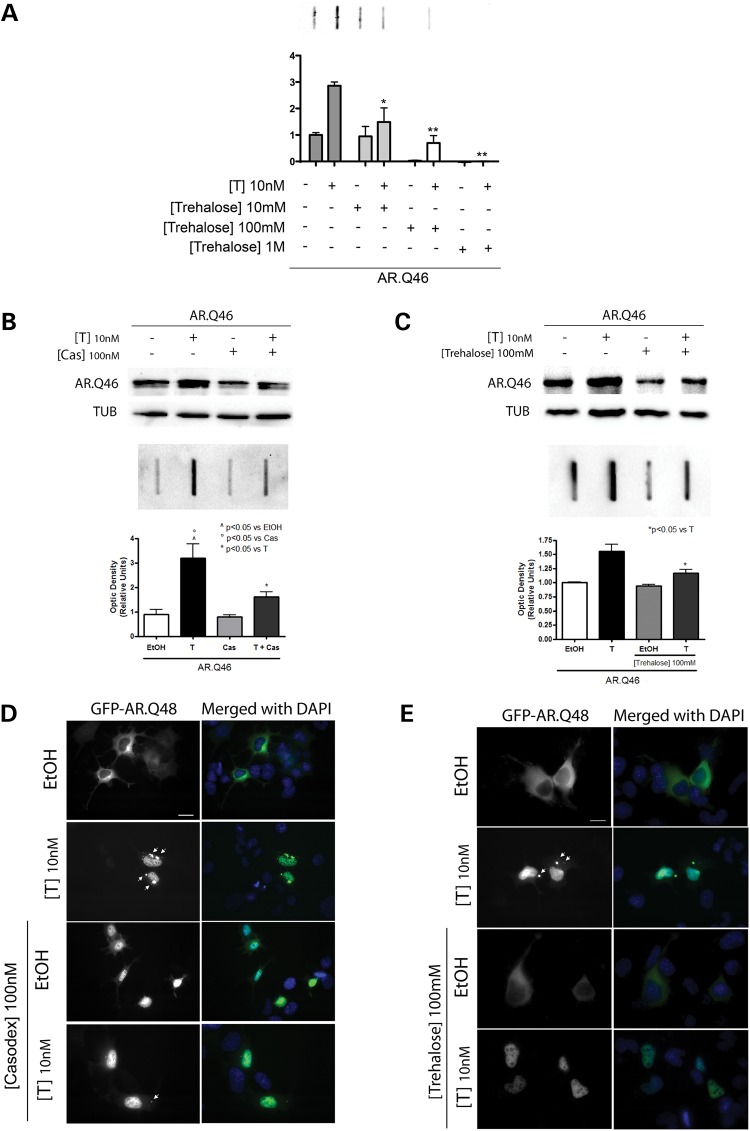
Effect of Bicalutamide or trehalose on testosterone-induced ARpolyQ accumulation. (**A**) NSC34 cells expressing AR.Q46 treated with ethanol (EtOH) as a vehicle control, 10 nm testosterone (T) in the absence of or in the presence of different doses of trehalose (10 mm/100 mm/1 m) for 48 h. FRA shows AR.Q46 insoluble species accumulation after T treatment, and the dose-dependent effects of trehalose (*P < 0.05 versus +T; **P < 0.001 versus +T). (**B**) NSC34 cells expressing AR.Q46 treated with ethanol (EtOH) as a vehicle control, 10 nm testosterone (T) and/or 100 nm Bicalutamide (Cas) for 48 h. (**C**) NSC34 cells expressing AR.Q46 treated with ethanol (EtOH) as a vehicle control or 10 nm testosterone (T), in the absence of or in the presence of 100 mm trehalose for 48 h. Western blot analysis (B and C, upper panels) shows soluble AR.Q46 protein levels following different treatments. Alpha-tubulin was used to normalize protein loading. FRA in B, lower panel, illustrates the different accumulation of insoluble species of AR.Q46 after T or/and Cas treatments (^^^*P* < 0.05 versus EtOH; °*P* < 0.05 versus +Cas; *P < 0.05 versus +T). FRA in C, lower panel, shows AR.Q46 insoluble species accumulation after T treatment, in the absence of or in the presence of trehalose (*P < 0.05 versus +T). (**D**) HRFM analysis (63× magnification) on NSC34 cells expressing GFP-AR.Q48 in the absence of (EtOH) or in the presence of 10 nm testosterone (T) and/or 100 nm Bicalutamide (Cas) for 48 h. Nuclei were stained with DAPI (blue). Scale bar = 10 μm. (**E**) HRFM analysis (63× magnification) on NSC34 cells expressing GFP-AR.Q48 in the absence of (EtOH) or in the presence of 10 nm testosterone (T), with or without 100 mm trehalose treatment for 48 h. Nuclei were stained with DAPI (blue). Scale bar = 10 μm.

In the first set of experiments performed using filter retardation assay (FRA), western blot (WB) and high-resolution fluorescence microscopy (HRFM) analysis, we compared the effects of single treatments with Bicalutamide or trehalose on ARpolyQ aggregation and clearance in the absence of or in the presence of testosterone (to trigger the formation of ARpolyQ toxic species) in NSC34 cells. The doses of trehalose have been selected by performing preliminary studies of dose-dependent response (Fig. 1A), while for Bicalutamide, which is a drug widely used in clinics against prostate cancer, and for which the pharmacokinetics is very well known, we used the doses classically adopted to counteract testosterone activity in all transcriptional and binding assays, as well as against ARpolyQ in SBMA (see, for example, 27,37,38). The results clearly demonstrate that Bicalutamide (Cas) reduces the accumulation of ARpolyQ insoluble species induced by testosterone (Fig. 1B, lower panel and relative quantification, FRA), and to a lesser extent the total levels of the monomeric ARpolyQ protein evaluated in western blot (Fig. 1B, upper panel, WB). This suggests that the misfolded ARpolyQ fraction, in particular, is targeted by the Bicalutamide prodegradative activity. Trehalose also significantly reduces the accumulation of mutant ARpolyQ insoluble species in FRA (Fig. 1C, lower panel and relative quantification), and reduces the monomeric ARpolyQ levels both in the absence of and in the presence of testosterone, as show in WB analysis (Fig. 1C, upper panel).

The presence of testosterone-induced ARpolyQ aggregates was analyzed both after Bicalutamide (Cas, Fig. 1D) and trehalose (Fig. 1E) treatment in HRFM analysis. It clearly appears that whereas large cytoplasmic aggregates of ARpolyQ are present in cells treated with testosterone, few and very small ARpolyQ aggregates are detectable in cells treated with either Bicalutamide (Cas) (no aggregates in Bicalutamide-treated cells in the absence of testosterone, approximately a 30% reduction induced by Bicalutamide on testosterone-induced ARpolyQ aggregates) or trehalose (no aggregates in trehalose-treated cells in the absence of testosterone, approximately a 50% reduction induced by trehalose on testosterone-induced ARpolyQ aggregates) in the presence of testosterone. In addition, Bicalutamide (Cas) greatly reduces nuclear translocation and accumulation of ARpolyQ, even in the presence of testosterone, retaining a large fraction of ARpolyQ in the cytoplasm. This effect is not present in trehalose-treated cells expressing ARpolyQ.

We next analyzed the effects of Bicalutamide and trehalose on two well-known autophagic markers, SQSTM1/p62 and LC3. The first, SQSTM1/p62, is upregulated during autophagy activation in our cell models and is responsible for insertion of polyubiquitinated misfolded protein species into autophagosomes; when autophagy is normally executed in cells, SQSTM1/p62 is continuously degraded via autophagy, together with the polyubiquitinated cargo that it recognizes and inserts into autophagosomes; conversely, when autophagy flux is blocked (i.e. by ARpolyQ), SQSTM1/p62 typically accumulates forming SQSTM1/p62 bodies that can be easily visualized in immunofluorescence (IF) microscopy. The second, LC3, is normally diffuse in cells as LC3-I in basal condition, but it is overexpressed during autophagy activation and converted into a lipidated autophagosome-anchored LC3-II protein showing a typical punctate distribution.

**Figure 2.**
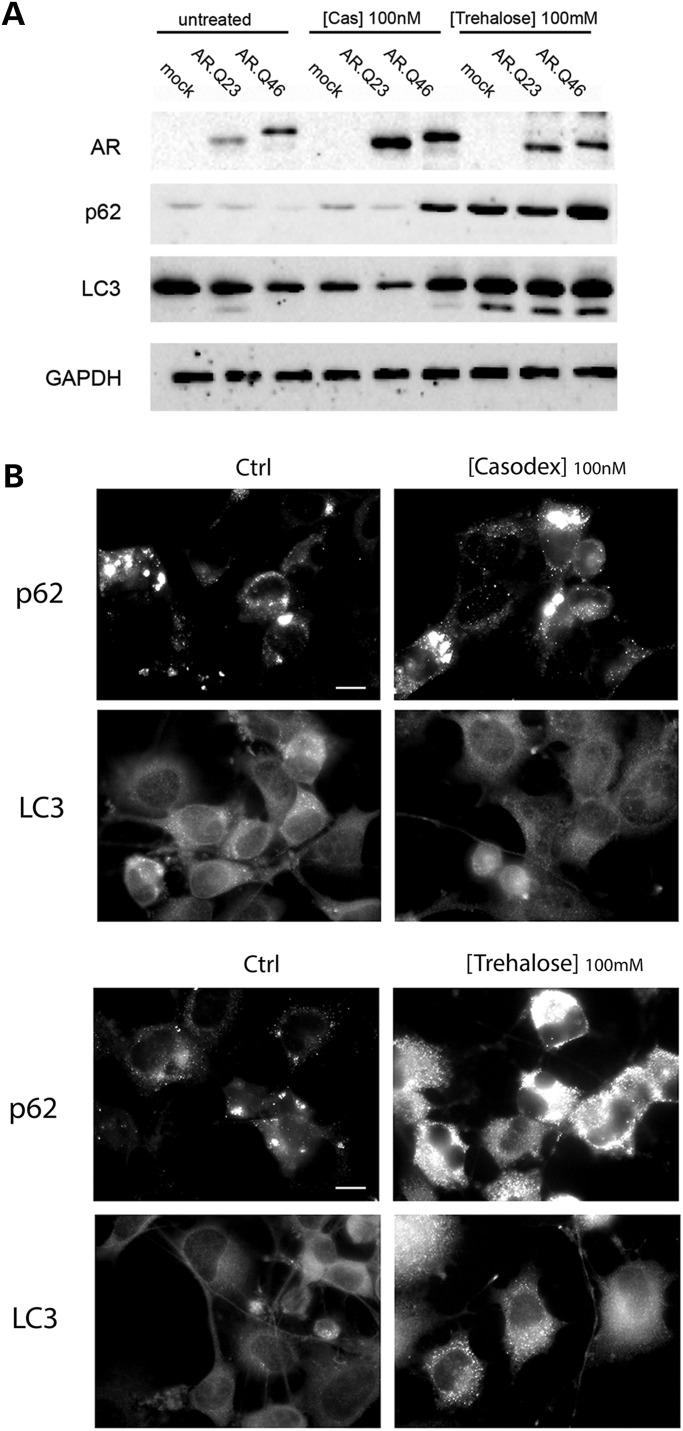
Autophagy activation is mediated by trehalose but not by Bicalutamide. (**A**) Western blot analysis on mock-transfected NSC34 cells and NSC34 cells transiently transfected with plasmids encoding AR.Q23 or AR.Q46 treated with 10 nm trehalose or 100 nm Bicalutamide (Cas) for 48 h. Two well-known markers of the autophagic pathway (SQSTM1/p62 and LC3-II) were used to compare the activation of autophagy mediated by trehalose or Bicalutamide. GAPDH was used to normalize protein loading. (**B**) HRFM analysis (63× magnification) on NSC34 cells shows the subcellular distribution and the endogenous expression levels of LC3 and SQSTM1/p62 after 100 nm Bicalutamide (upper panel) or 100 mm trehalose (lower panel) treatments for 48 h, in the presence of mutant AR. Scale bar = 10 μm.

WB analysis in Figure 2A shows that SQSTM1/p62 levels remains unchanged after Bicalutamide treatment, but are increased by trehalose both in wild-type (wt; AR.Q23) and in ARpolyQ (AR.Q46) expressing cells. Similarly, Bicalutamide has no effect on LC3-I to LC3-II conversion, whereas trehalose stimulates the formation of large amounts of LC3-II form. These data were confirmed in IF microscopy (Fig. 2B), where it is noteworthy that SQSTM1/p62 and LC3 protein expression levels were greatly increased after trehalose treatment, but not by Bicalutamide.

**Figure 3.**
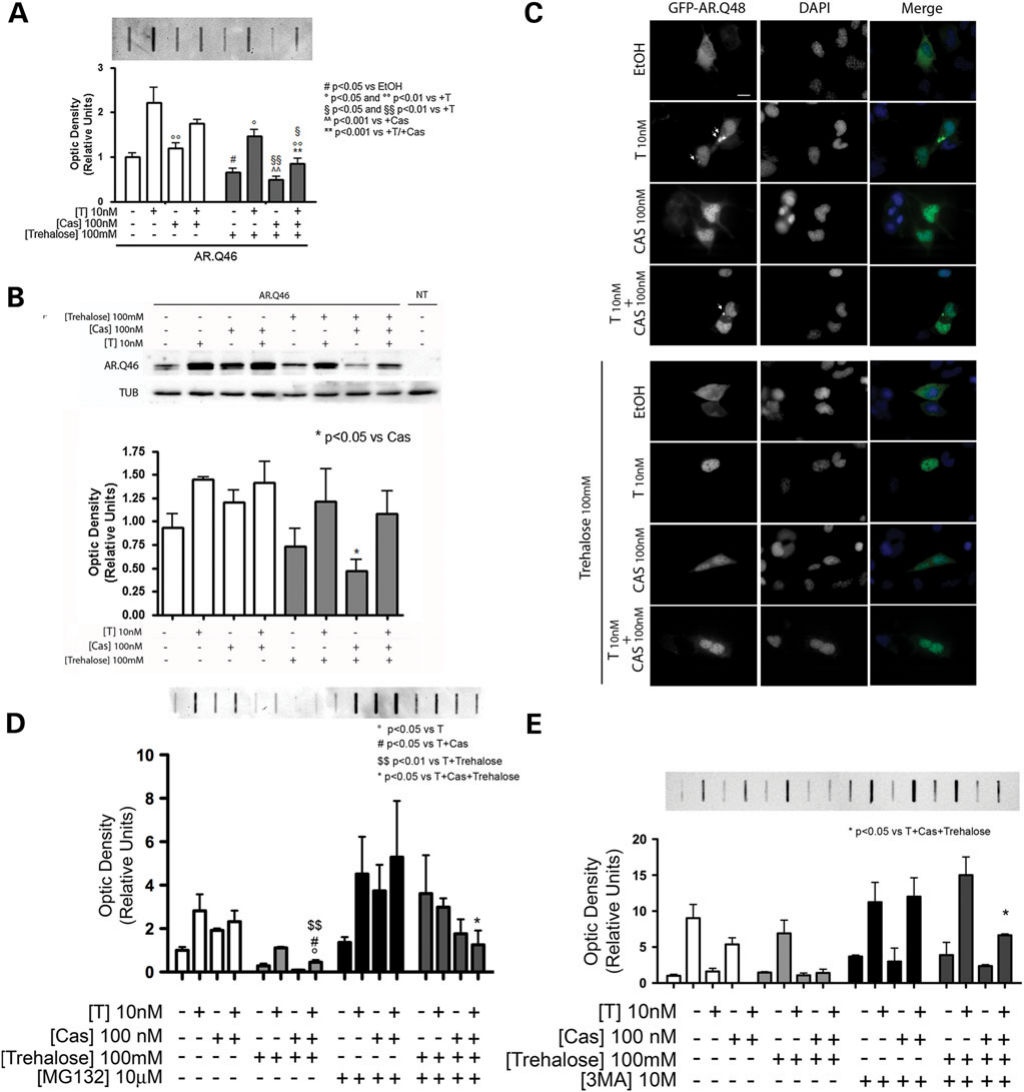
Combined effect of Bicalutamide and trehalose on AR.Q46 accumulation. (**A** and **B**) NSC34 cells expressing AR.Q46 treated with ethanol (EtOH) as a vehicle control, 10 nm testosterone (T) and/or 100 nm Bicalutamide (Cas) and/or 100 mm trehalose for 48 h. FRA (A) shows the synergic effect of Bicalutamide and trehalose on the accumulation of insoluble species of AR.Q46. The histogram represents a quantitative evaluation of AR.Q46 protein level carried out by densitometric scanning of the blots (^#^*P* < 0.05 versus EtOH; °*P* < 0.05 and °°*P* < 0.01 versus +T; ^§^*P* < 0.05 and ^§§^*P* < 0.01 versus +T; ^^^^*P* < 0.001 versus +Cas; **P < 0.001 versus +T/+Cas). Western blot analysis (B) displays soluble AR.Q46 expression levels after single or combined treatments. The histogram represents a quantitative evaluation of AR.Q46 protein level normalized on alpha-tubulin carried out by densitometric scanning of the blots (*P < 0.05 versus +Cas). (**C**) HRFM analysis (63× magnification) on NSC34 cells expressing GFP-AR.Q48 in the absence of (EtOH) or in the presence of 10 nm testosterone (T) and/or 100 nm Bicalutamide (Cas), with or without 100 mm trehalose treatment for 48 h. Nuclei were stained with DAPI (blue). Scale bar = 10 μm. (**D**) FRA on NSC34 cells expressing AR.Q46 treated with ethanol (EtOH) as a vehicle control, 10 nm testosterone (T) and/or 100 nm Bicalutamide (Cas) and/or 100 mm trehalose for 48 h. AR.Q46 protein accumulation was analyzed in the condition described above or after treatment with 10 μm of MG132 for 16 h to inhibit the proteasome. The histogram represents a quantitative evaluation of AR.Q46 protein level carried out by densitometric scanning of the blots (°*P* < 0.05 versus T; ^#^*P* < 0.05 versus T + Cas; ^$$^*P* < 0.01 versus T + trehalose; *P < 0.05 versus T + Cas + trehalose). (**E**) FRA on NSC34 cells expressing AR.Q46 treated with ethanol (EtOH) as a vehicle control, 10 nm testosterone (T) and/or 100 nm Bicalutamide (Cas) and/or 100 mm trehalose for 48 h. AR.Q46 protein accumulation was analyzed in the condition described above or after treatment with 10 mm of 3-MA for 48 h to block the autophagosome formation. The histogram represents a quantitative evaluation of AR.Q46 protein level carried out by densitometric scanning of the blots (*P < 0.05 versus T + Cas + trehalose).

We next analyzed the possible synergic effect of the combinatory use of Bicalutamide and trehalose on the ARpolyQ solubility in immortalized motoneuronal cells, both in the absence of and in the presence of testosterone. FRA analysis (Fig. 3A) clearly demonstrates that the levels of testosterone-induced ARpolyQ insoluble species detected in cells simultaneously treated with Bicalutamide (Cas) and trehalose are significantly lower than that found in Bicalutamide (Cas)-treated cells (*P* < 0.001) or in trehalose-treated cells (*P* < 0.05). A similar effect is also present in ARpolyQ insoluble species accumulating in the absence of testosterone, but in this case, the total insoluble material found in FRA is much lower than that found in testosterone-treated cells.

We also analyzed the effect of the combined treatment with Bicalutamide and trehalose on monomeric ARpolyQ in WB. Figure 3B shows that, in this case, the combined treatment with Bicalutamide (Cas) and trehalose results in a mild reduction of ARpolyQ monomeric species, which is not significantly different from that observed after the separate treatments with the two compounds. Thus, the combinatory use of Bicalutamide and trehalose mainly affects the insoluble aggregated fraction derived from the misfolded ARpolyQ and not the monomeric, possibly normally folded, ARpolyQ present in immortalized motoneuronal cells.

The presence of testosterone-induced aggregates of ARpolyQ was evaluated in HRFM analysis (Fig. 3C). We found that the combined treatment with Bicalutamide (Cas) and trehalose completely prevents the formation of ARpolyQ aggregates (no aggregates in trehalose-treated or Bicalutamide-treated cells in the absence of testosterone, approximately a 80% reduction induced by Bicalutamide and trehalose co-treatment on testosterone-induced ARpolyQ aggregates).

Since the UPS, together with autophagy, is involved in the removal of ARpolyQ misfolded species, we analyzed whether UPS inhibition prevents the prodegradative activity of the combined treatment with Bicalutamide and trehalose. The FRA data reported in Figure 3D confirm the efficacy of the two drugs used in combination in the absence of MG132, which inhibits the chimotryptic activity of the proteasome. In addition, the data suggest that the prodegradative activity exerted by Bicalutamide on ARpolyQ mainly requires a robust proteasomal activity; in fact, a large fraction of misfolded ARpolyQ appears when the UPS is inhibited with MG132, even in the presence of Bicalutamide (Cas). Trehalose, which is an autophagy inducer, tends to increase insoluble ARpolyQ clearance also in the presence of MG132, but not in a significant manner. The combined treatment with Bicalutamide (Cas) and trehalose further increases the clearance of insoluble ARpolyQ, even in the presence of MG132. The role of the autophagic pathway was analyzed by blocking the autophagic flux with 3-methyladenine (3-MA), which prevents the assembly of mature autophagosomes. The data shown in Figure 3E clearly indicate that autophagy inhibition results in a complete loss of the prodegradative synergistic activity of Bicalutamide (Cas) and trehalose on ARpolyQ insoluble species. Moreover, with single Bicalutamide or trehalose treatment, autophagy blockage by 3-MA results in a mild accumulation of mutant AR species, but not statistically relevant. Both the effects of single and double treatments are counteracted by autophagy inhibition, indicating that autophagy is active together with the ubiquitin–proteasome degradative system. Therefore, although the UPS is largely involved in the removal of misfolded insoluble ARpolyQ, cytoplasmic retention of the ARpolyQ, combined with the activation of the cytoplasmic autophagic process, efficiently prevents its accumulation in cells.

**Figure 4.**
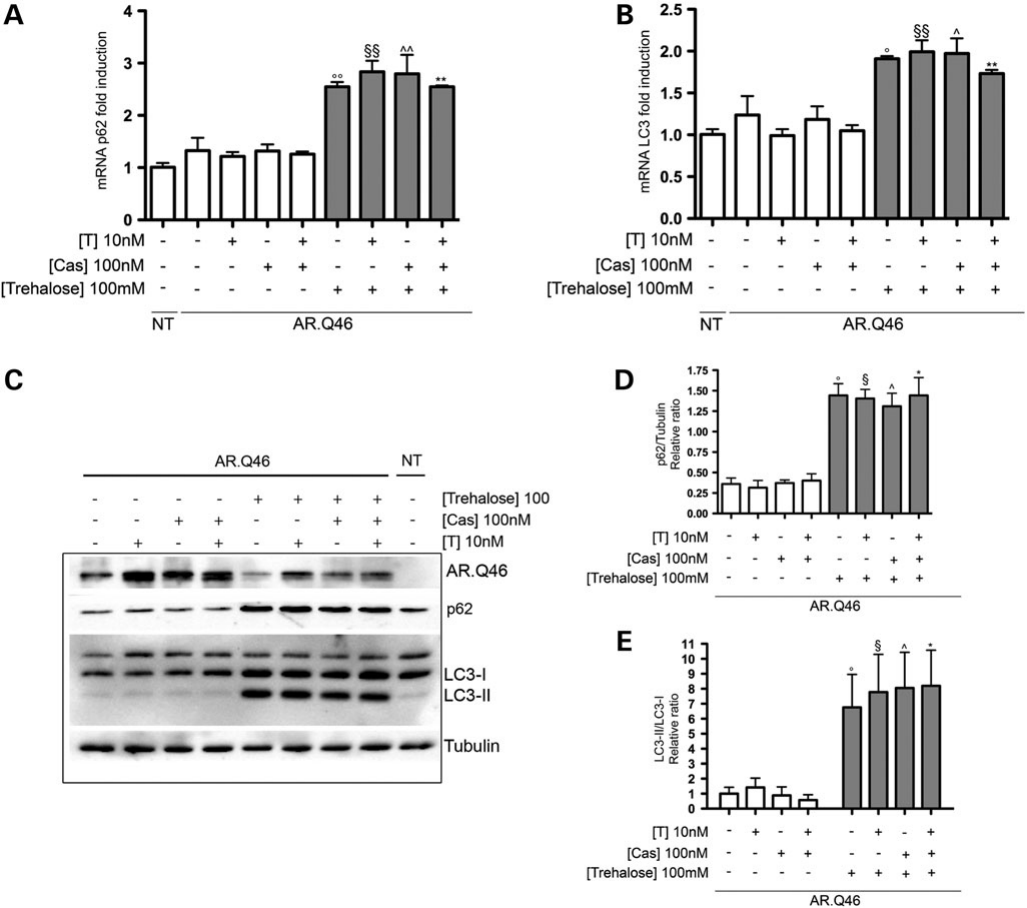
The autophagy activation is mediated by trehalose and not influenced by Bicalutamide. (**A** and **B**) Real-time PCR on SQSTM1/p62 (A) and on LC3 (B) mRNA expression levels on NSC34 cells expressing AR.Q46, in the absence of (ethanol as a vehicle control) or in the presence of 10 nm testosterone (T) and/or 100 nm Bicalutamide (Cas) and/or 100 mm trehalose for 48 h (°°*P* < 0.01 versus EtOH; ^§§^*P* < 0.01 versus +T; ^^*P* < 0.01 versus +Cas; **P < 0.01 versus +T/+Cas). Not transfected cells (NT) show that AR.Q46 transfection does not alter SQSTM1/p62 and LC3 mRNA expression levels. (**C**) Western blot analysis on NSC34 cells expressing AR.Q46 treated with ethanol (EtOH) as a vehicle control, 10 nm testosterone (T) and/or 100 nm Bicalutamide (Cas) and/or 100 mm trehalose for 48 h. Two well-known markers of the autophagic pathway (SQSTM1/p62 and LC3-II) were used to show that the activation of autophagy is mediated by trehalose and not influenced by Bicalutamide. NT demonstrates that AR.Q46 transfection does not influence SQSTM1/p62 and LC3-II protein expression levels. Alpha-tubulin was used to normalize protein loading. (**D** and **E**) The two histograms, relative to western blot analysis in (C), represent a quantitative evaluation of SQSTM1/p62 protein level normalized on alpha-tubulin (D) (°*P* < 0.05 versus EtOH; ^§^*P* < 0.05 versus +T; ^^^*P* < 0.05 versus +Cas; *P < 0.05 versus +T/+Cas) and a quantitative evaluation of LC3-II/LC3-I ratio protein level (E) (°*P* < 0.05 versus EtOH; ^§^*P* < 0.05 versus +T; ^^^*P* < 0.05 versus +Cas; *P < 0.05 versus +T/+Cas), carried out by densitometric scanning of the blots.

Based on these data we wished to evaluate the involvement of the autophagic process in the combined activity of Bicalutamide (Cas) and trehalose. Thus, we analyzed SQSTM1/p62 and LC3 expression and processing. As shown in Figure 4A, trehalose, alone or in combination with Bicalutamide (Cas), significantly increases SQSTM1/p62 mRNA expression levels in our cells expressing ARpolyQ; Bicalutamide (Cas) is unable to enhance SQSTM1/p62 mRNA expression levels and does not amplify the trehalose-stimulated SQSTM1/p62 expression, both in the absence and presence of testosterone. Overlapping results were obtained when, in the same conditions, we analyzed the LC3 mRNA expression levels (Fig. 4B). In addition, the levels of the SQSTM1/p62 protein and the conversion of LC3-I to its lipidated and autophagosome-associated form, LC3-II, were increased after trehalose treatment but not influenced by the co-treatment with Bicalutamide (Fig. 4C and relative quantifications for SQSTM1/p62 in D and for LC3-II/LC3-I ratio in E).

**Figure 5.**
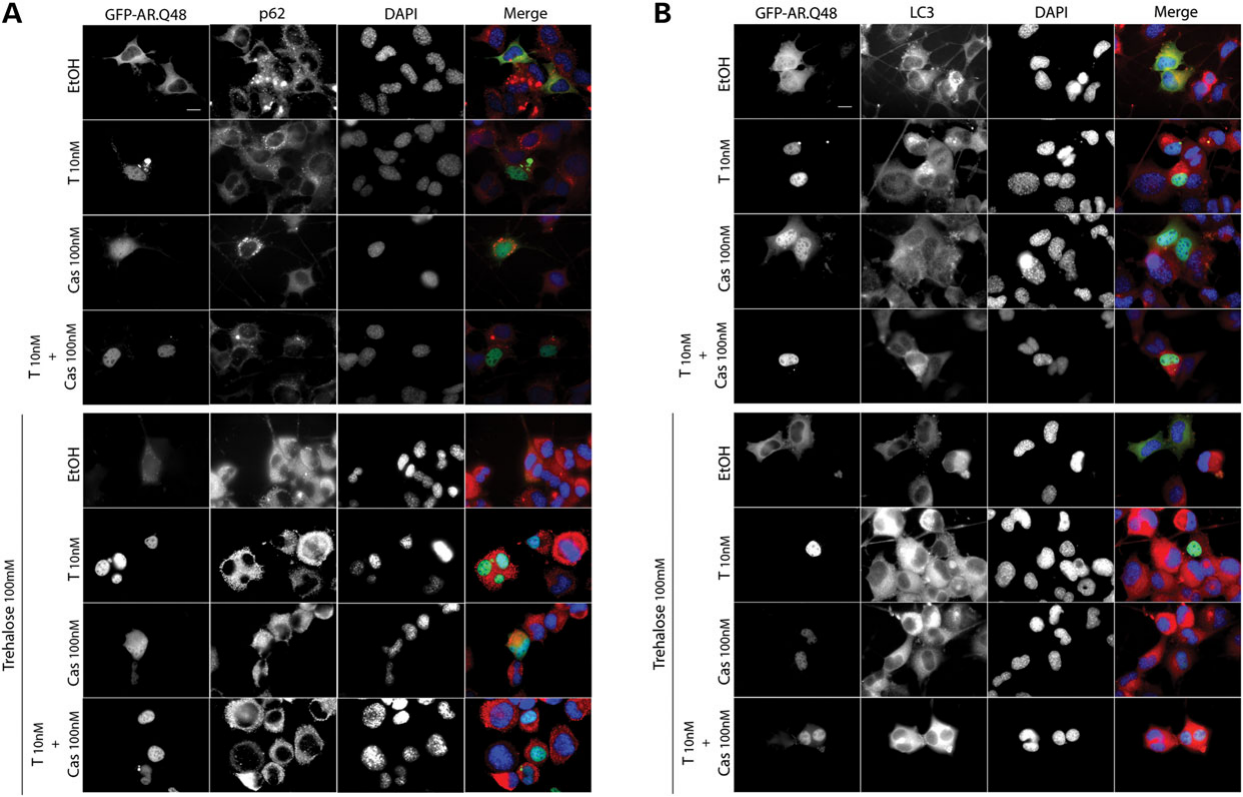
SQSTM1/p62 and LC3 distribution. (**A** and **B**) HRFM analysis (63× magnification) on NSC34 cells expressing GFP-AR.Q48 in the absence of (EtOH) or in the presence of 10 nm testosterone (T) and/or 100 nm Bicalutamide (Cas), with or without 100 mm trehalose treatment for 48 h. Endogenous SQSTM1/p62 (A) and LC3 (B) protein distribution and expression levels are shown in red. Nuclei were stained with DAPI (blue). Scale bar = 10 μm.

These results were further supported by the data obtained in IF microscopy analysis (Fig. 5) When cells were not treated with trehalose, SQSTM1/p62 shows an irregular and disorganized distribution, as shown in Figure 5A. In the presence of trehalose, SQSTM1/p62 expression levels notably increase and SQSTM1/p62 is homogeneously distributed into the entire cell cytoplasm. No changes in SQSTM1/p62 expression and distribution are induced by Bicalutamide (Cas) treatment. In the case of LC3 distribution analyzed in IF (Fig. 5B), we found that Bicalutamide (Cas) does not influence the levels or the overall punctate distribution of LC3-II induced by mutant ARpolyQ (both in the absence and presence of testosterone); conversely, trehalose treatment, which stimulates autophagy, enhances LC3-II levels which become exclusively present in its punctate distribution. As expected, similar levels and distribution of LC3-II are present in immortalized motoneurons expressing ARpolyQ and simultaneously treated with Bicalutamide (Cas) and trehalose (both in the absence and presence of testosterone). In all cases, Bicalutamide and trehalose alone or in combination are all capable to reduce the number of ARpolyQ aggregates formed after testosterone treatment. All together, the data strongly suggest the presence of active autophagy capable to remove the cytoplasmically retained ARpolyQ.

**Figure 6.**
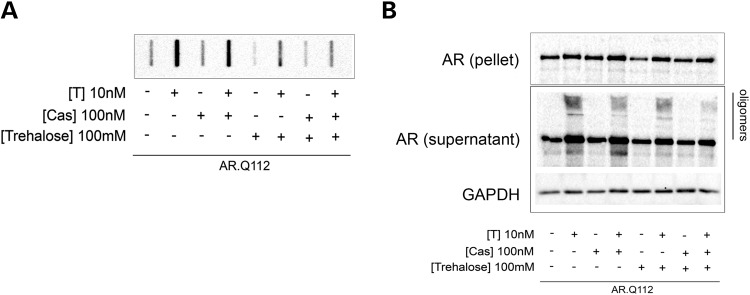
Combined effect of Bicalutamide and trehalose on PC12 cells expressing AR.Q112. (**A** and **B**) AR.Q112 protein expression in PC12 stable transfected cell line was induced by 1 μg/ml of doxycycline; after 12 h the cells were treated with ethanol (EtOH) as a vehicle control, 10 nm testosterone (T) and/or 100 nm Bicalutamide (Cas) and/or 100 mm trehalose for 48 h. The cells were re-suspended in RIPA buffer and centrifuged to separate the pellet and the supernatant fractions. The supernatant fraction was loaded in FRA (A). Both the supernatant and the pellet fractions were analyzed by western blot (B). GAPDH was used to normalize protein loading.

To further characterize the effects of the combinatory use of Bicalutamide and trehalose, their activity was also tested on a form of AR containing a very long polyQ (Q112) tract, which has a different kinetic of aggregation and generates nuclear inclusions. To accomplish this, we used the PC12/AR.Q112 TET-On inducible cell model of SBMA. We demonstrated in FRA (Fig. 6A) that both Bicalutamide (Cas) and trehalose decrease the ARpolyQ(112) aggregation, but the combinatory use of the two compounds is far more efficient than the two compounds used singularly. A similar effect was observed on the insoluble fraction (pellet) recovered after high-speed centrifugation of cell lysates of PC12/AR.Q112 cells (Fig. 6B). These results demonstrate a reduction in the amount of ARpolyQ(112) present in the pellet fraction from cells treated simultaneously with Bicalutamide and trehalose (in the presence of testosterone). We also observed a trend toward a reduction in the levels of the monomeric form of ARpolyQ in the supernatant fractions, but these data did not reach statistical significance (Fig. 6B). This may reflect the possibility that the insoluble aggregated forms of ARpolyQ (more than the soluble monomeric forms of ARpolyQ) are preferentially degraded after the combinatory use of Bicalutamide and trehalose. Moreover, it is noteworthy that there is a reduction in the amount of ARpolyQ oligomers, observed in the stacking gel, due to the combined treatment.

## DISCUSSION

In this study, we evaluated whether a combined pharmacological treatment, aimed both to reduce the nuclear toxicity exerted by mutant AR and to enhance its autophagic clearance, may be of use as a novel therapeutic approach in SBMA (21,30,34,50). Our data clearly demonstrate that the simultaneous use of Bicalutamide (which reduces the rate of AR nuclear translocation) (27,28,37,38,49) and trehalose (an autophagy activator and HSPB8 stimulator) (37,43,44,46,48) results in an increase in the clearance of insoluble ARpolyQ species induced by testosterone treatment in immortalized motoneuronal cells. Here, we decided to focus our attention only on motoneuronal cells, since the molecular events at the basis of ARpolyQ neurotoxicity are quite well studied, while the muscle-related toxicity, which is clearly exerted by the SBMA ARpolyQ, is still poorly understood. In any case, aberrations in both cell types participate to the onset and progression of the disease, even if it is still debated the relative contribution of each cell type to SBMA. It remain to be determined whether the therapeutic approach here postulated may have beneficial effects also in muscle, in which the toxicity exerted by ARpolyQ may be due to different causes. We believe that further studies on muscle cell models and a trial on SBMA mice models may provide the answer to this question.

In general, the combined treatment has no relevant effects on the levels of monomeric soluble ARpolyQ, both in the absence and presence of testosterone; in fact, the prodegradative effect of the two compounds acting in an additive manner is mainly exerted on the ARpolyQ misfolded fraction. It is this fraction that is capable of generating insoluble species and forming intracellular aggregates. Our data suggest that, at least in their earlier formation stages, aggregates may protect against ARpolyQ toxicity (39,41) by confining neurotoxic species into a physically defined subcellular compartment. However, they may become toxic at later stages by altering essential neuronal processes (17,40). In any case, aggregates represent a valuable marker to follow protein misfolding, and the reduction in their number is indicative of a beneficial effect of the combinatory treatment against misfolded species in affected cells. Indeed, in this study, we show that ARpolyQ aggregates were more efficiently reduced by the combined action of Bicalutamide and trehalose than when the same compounds were used separately. Therefore, the longer cytoplasmic retention of the Bicalutamide-bound ARpolyQ facilitates a better cytoplasmic autophagic recognition of the misfolded species prior to its migration into the nucleus; at the same time, the trehalose-enhanced autophagy improves the capability of this proteolytic system to clear the excess of misfolded ARpolyQ released from accessory chaperones after ligand interaction, also preventing any possible autophagic flux blockage. This can be also achieved by trehalose induction of the small HSPB8 (37), which we have previously shown to be a facilitator of autophagy. In fact, the response of cells to protein misfolding requires different HSPs (28,36,51,52). HSPB8 dramatically increases solubility and clearance of ARpolyQ (and other misfolded proteins), decreasing their aggregation rate (37,53–55). HSPB8 acts via both the UPS and autophagy (37,54,55); HSPB8 facilitation of autophagy requires the formation of a complex with BAG3/HSC70/CHIP, in which CHIP ubiquitinates HSPB8-associated substrates, allowing SQSTM1/p62 recognition for insertion into autophagosomes (54). With this mechanism, HSPB8 facilitates ARpolyQ autophagic clearance and relieves the blockage of autophagic flux (37).

Several advantages may arise from the use of these two active compounds in SBMA. First, the antiandrogen Bicalutamide is an FDA-approved and commercially available drug (Casodex), already widely used in prostate cancer therapy. It can be administered for chronic treatment and is relatively well tolerated by patients. Secondly, trehalose is a natural disaccharide widely present in microorganisms, plants and invertebrates, and used in several nutrients to preserve aliments. It is a nontoxic and very well-tolerated molecule consisting of two d-glucoses connected by an α,α-1,1-linkage. Trehalose is not considered a drug, but a nutrient supplement, with no particular restriction for its oral intake. Trehalose is hydrolyzed to glucose within the gastrointestinal tract, but the fraction adsorbed can reach the brain (see below). Trehalose was initially found active in a screening designed to find inhibitors of polyQ-mediated protein aggregation and was tested with various disaccharides because of the lack of toxicity and the possibility of safe, oral administration (43). Trehalose has been proved active against aggregation of polyQ-expanded huntingtin (43,44), beta-amyloid (36,42,56), polyA-binding protein nuclear 1 (PABPN1) (45), the pathological prion protein (46), the amyotrophic lateral sclerosis (ALS) and frontotemporal lobar degeneration (FTLD)-associated protein TDP-43 (57), mutant synuclein (47) and also on the ARpolyQ (37). Interestingly, tested in animal models, trehalose alone has been found useful in several mouse models of NDs. For example, the oral administration of trehalose counteracted polyQ toxicity and ameliorated the phenotype, improving motor dysfunction and extended life span in a mouse model of Huntington disease (HD) (43); trehalose administration resulted in a decrease of aggregation of huntingtin mutant protein in several brain areas (but also in liver), thus even when administered orally, it can be adsorbed and a fraction non metabolized in the gastrointestinal tract, can cross the brain blood barrier reaching the neuronal cells affected in HD (43). In mouse models of oculopharyngeal muscular dystrophy (OPMD), linked to an abnormal expansion of a polyalanine tract in PABPN1, oral administration of trehalose attenuated muscle weakness and reduced mutant PABPN1 aggregation in skeletal muscle (45). Two recent reports have demonstrated that trehalose also improves motor function and extends survival of mouse models of ALS (42,48). These data together demonstrate that trehalose is a good candidate molecule to be used in future therapeutic approaches in human NDs. Unfortunately, even if trehalose has been found active in cellular models of SBMA (37,49), no studies have been conducted in mouse models of SBMA. Similarly, several studies performed in cellular models of SBMA have demonstrated that Bicalutamide alone counteracts ARpolyQ aggregation (37,38) possibly facilitating autophagic removal of ARpolyQ misfolded species, thus preventing its neurotoxicity. Even in this case, no data are yet available in mouse models of SBMA.

In this study, we tested the combinatory use of Bicalutamide and trehalose on cell models of SBMA, demonstrating a synergistic effect of the two compounds on ARpolyQ removal. The slowed kinetics of ARpolyQ nuclear translocation induced by Bicalutamide allows for an increased clearance of misfolded ARpolyQ species via the cytoplasmic autophagic machinery, rendered more efficient and active by trehalose treatment. It is worth noting that the autophagic process is induced by trehalose but not influenced by Bicalutamide, as shown by the two well-known autophagic markers, SQSTM1/p62 and LC3-II. Collectively, the data here reported lay the foundation for preclinical trials in mouse models of SBMA to test the therapeutic potential of Bicalutamide and trehalose in single or combined administration.

## MATERIALS AND METHODS

Chemicals, MG-132, 3-MA and trehalose have been obtained from Sigma-Aldrich (St. Louis, MO, USA).

### Plasmids

The plasmids coding for AR.Q23 and AR.Q46, routinely used in our laboratory have been previously described (39). The plasmid coding for the protein GFP-AR.Q48 was obtained by insertion of AR cDNA into the Green Fluorescent Protein (GFP) vector, expressing chimeric fluorescent fusion proteins.

### Cell cultures and transfections

The immortalized motoneuronal cell line NSC34 (58,59) is routinely used in our laboratory (24,37,39–41,52,54,60–62) and has been transfected with Lipofectamine (Life Technologies Corporation, Carlsbad, CA, USA)/transferrin (Sigma-Aldrich), as previously described (39,40,60), using 0.6 µg of plasmid DNA, 4 µl of transferrin solution and 2 µl of Lipofectamine.

Rat adrenal pheochromocytoma (PC12) cells stable transfected with the plasmid encoding AR.Qn (n010, 112) express AR under the control of a Tet-On promoter responsive to 1 µg/ml of doxycycline [produced (and kindly provided) by Professor D. Merry (TJU, Philadelphia, PA, USA)]. In the experiments involving steroid hormone treatments, the fetal bovine serum (FBS) was replaced with charcoal-stripped FBS and the horse serum (HS) with charcoal-stripped HS, to eliminate endogenous steroids (24,40,63).

### mRNA expression analysis

NSC34 cells were plated at 80 000 cells/ml in six-well multiwell plates, transfected as described above and treated with 100 mm trehalose and/or 100 nm Bicalutamide and/or 10 nm testosterone for 48 h.

Forty-eight hours after transfection, cells were harvested and centrifuged 5 min at 100 *g* at 4°C; the pellets were resuspended in 300 μl of TRI Reagent (Sigma-Aldrich) and RNA isolated according to manufacturer's instruction. RNA quantification was carried out by absorbance at 260 nm. Total RNA (1 μg) was treated with DNAse (Sigma-Aldrich), and reverse transcribed into cDNA using the High-Capacity cDNA Archive Kit (Life Technologies Corporation) according to the manufacturer's protocol. Primers for real-time RT-PCR of the LC3-B, SQSTM1/p62 and GAPDH mRNAs were designed using the program Primer 3. The primers were synthesized by MWG Biotech (Ebersberg, Germany) with the following sequence: *MAP-LC3b*: 5′- CGT CCT GGA CAA GAC CA-3′ (forward), 5′-CCA TTC ACC AGG AGG AA-3′ (reverse); *SQSTM1/p62:* 5′-AGG GAA CAC AGC AAG CT-3′ (forward), 5′-GCC AAA GTG TCC ATG TTT CA-3′ (reverse); *GAPDH*: 5′-CCA GAA CAT CAT CCC TGC AT-3′ (forward), 5′-CAG TGA GCT TCC CGT TCA-3′ (reverse). The evaluated efficiency of each set of primers was close to 100% for both target and reference genes. Real-time PCR was performed using the CFX 96 Real-Time System (Bio-Rad, Hercules, CA, USA) in a 10 µl total volume, using the iTaq SYBR Green Supermix (Bio-Rad), and with 500 nm primers. PCR cycling conditions were as follows: 94°C for 10 min, 40 cycles at 94°C for 15 s and 60°C for 1 min. Melting curve analysis was performed at the end of each PCR assay as a control for specificity. Data were expressed as *C*_t_ values and used for the relative quantification of targets with the ΔΔ*C*_t_ calculation. To exclude potential bias due to averaging data transformed through the equation $2^{-\Delta \Delta C_{t}}$ to give *N*-fold changes in gene expression, all statistics were performed with Δ*C*_t_ values. Each experiment was carried out with four independent samples.

### Western blot (WB) analysis and FRA

NSC34 cells were plated in 12-well multiwell plates at 80 000 cell/ml, transfected as previously described. For dose-dependent analysis, the cells were treated with 10 nm/100 nm/1 µm Bicalutamide or 10 mm/100 mm/1 M trehalose. In the other experiments, the cells were treated with 100 mm trehalose and/or 100 nm Bicalutamide and/or 10 nm testosterone for 48 h. In experiments involving autophagy blockage, 10 mm 3-MA was added to the cells for the last 48 h. In experiments where proteasome was inhibited, the cells were treated with 10 µm MG132 for the last 16 h (overnight treatment).

After 48 h, cells were harvested and centrifuged 5 min at 100 *g* at 4°C; the cell pellets were re-suspended in PBS (added of the protease inhibitors cocktail, Sigma-Aldrich) and homogenized using slight sonication as previously described (63). Total proteins were determined with the bicinchoninic acid method (BCA assay, Euroclone).

PC12/AR.Q112 cells were plated in six-well multiwell plates at 80 000 cell/ml, induced with 1 μg/ml of doxycycline and treated with 100 mm trehalose and/or 100 nm Bicalutamide and/or 10 nm testosterone for 48 h. After 48 h, cells were harvested and centrifuged 5 min at 100 *g* at 4°C; the cell pellets were re-suspended in RIPA buffer (added of the protease inhibitors cocktail, Sigma-Aldrich) and homogenized using slight sonication. The samples were centrifuged 15 min at 15 000 *g* at 4°C. The soluble fraction (supernatant) was separated from the insoluble one and the protein concentration was determined by the BCA assay. The pellet fraction was re-suspended in 20 μl of 4x sample buffer, and a relative amount was loaded into the gel, according to the protein assay on the soluble fraction.

WB was performed on 15% SDS–polyacrylamide gel electrophoresis loading 10–15 µg of total proteins. Samples were then electrotransferred to PVDF (polyscreen transfer membrane, PerkinElmer, Waltham, MA, USA) using a semi-dry transfer apparatus (Trans-Blot^®^ Turbo™ Transfer System, Bio-Rad). The membranes were treated with a blocking solution containing 5% nonfat dried milk powder (EuroClone, MI, Italy) in Tris buffered saline with Tween for 1 h and then incubated with the following primary antibodies: (a) rabbit polyclonal AR-H280 (dilution 1 : 3000) to detect wt AR and ARpolyQ; (b) rabbit polyclonal anti-LC3 (dilution 1:4000) to detect LC3; (c) rabbit polyclonal anti-SQSTM1/p62 (dilution 1:4000) to detect SQSTM1/p62; (d) mouse monoclonal anti-α-tubulin (Sigma-Aldrich, dilution 1:4000) to detect total α-tubulin. Immunoreactivity was detected using the following secondary peroxidase-conjugated antibodies: goat anti-rabbit (Santa Cruz, dilution 1:20 000) was used to identify the anti-AR, the anti-LC3 and the anti-SQSTM1/p62 antibodies; goat anti-mouse (sc-2005, Santa Cruz, dilution 1:20 000) was used to identify the anti-α-tubulin antibody. The immunoreactive regions were then visualized using the enhanced chemiluminescence detection kit reagents (ECL prime Western Blotting Substrate, GE Healthcare, Maidstone, UK). The same membranes were subsequently processed with different antibodies to detect the levels of different proteins in the same sample loaded on the gel, after stripping for 10 min at room temperature (StripABlot, EuroClone).

FRA was performed by sample filtration through a 0.2 µm cellulose acetate membrane (Whatman, GE Healthcare) using a Bio-Dot SF Microfiltration Apparatus (Bio-Rad) and loading 9 µg (NSC34 cells) of the total proteins or 1.5 µg (PC12) of the supernatant fraction. Slot-blots were probed as described for WB to detect AR.Q46 or AR.Q112.

A ChemiDoc XRS System (Bio-Rad) was used for the image acquisition of WB and FRA. Optical intensity of samples assayed with WB or FRA was detected and analyzed using the Image Lab software (Bio-Rad).

### Fluorescence, IF and microscopy on NSC34 cells

NSC34 cells were plated in 12-well multiwell plates containing coverslips at 70 000 cells/well density, transiently transfected with the plasmid coding for GFP-ARQ48 as previously described and treated with 100 mm trehalose and/or 100 nm Bicalutamide and/or 10 nm testosterone for 48 h. The cells were then fixed and processed as previously described (Sau, 2007). To analyze LC3 and SQSTM1/p62 protein expressions, we used the following primary antibodies: rabbit polyclonal anti-LC3-B antibody 1 : 1000 (Sigma-Aldrich) in 5% nonfat milk, rabbit polyclonal anti-SQSTM1/p62 antibody 1 : 1000 (Abcam, Cambridge, UK) in 5% nonfat milk. Secondary antibodies used were: Alexa 488 anti-rabbit and Alexa 594 anti-rabbit (Life Technologies) 1 : 1000 in milk. Cells were stained with DAPI to visualize the nuclei. An Axiovert 200 microscope (Zeiss Instr., Oberkochen, Germany) equipped with FITC/TRITC/DAPI and combined with a Photometric Cool-Snap CCD camera (Ropper Scientific, Trenton, NJ, USA) was used. Images were processed using the Metamorph software (Universal Imaging, Downingtown, PA, USA).

### Statistical analysis

Statistical analysis has been performed using one-tailed Student's *t*-test for two group comparisons and one- or two-way ANOVA for three or more group comparisons using the PRISM software (GraphPad Software, La Jolla, CA, USA).

*Conflict of Interest statement*. None declared.

## FUNDING

This work was supported by Telethon—Italy (GGP07063 and GGP14039) to A.P.; AriSLA Foundation Italy (ALS_HSPB8) to A.P.; Italian Ministry of Labour, Health and Social Affairs (Convenzione Fondazione Mondino/UNIMI) to A.P. and (Giovani Ricercatori) to V.C.; Regione Lombardia to A.P.; Universita’ degli Studi di Milano to A.P. and P.R.; Fondazione CARIPLO (2008-2307) to A.P.; Fondation Thierry Latran, France (FTL n. AAP091102) to A.P., Association Française contre les Myopathies (project n. 16406) to A.P. Funding to pay the Open Access publication charges for this article was provided by Fondazione Telethon, Italy.

## References

[1] Kennedy W.R., Alter M., Sung J.H. (1968). Progressive proximal spinal and bulbar muscular atrophy of late onset. A sex-linked recessive trait. Neurology.

[2] Sobue G., Hashizume Y., Mukai E., Hirayama M., Mitsuma T., Takahashi A. (1989). X-linked recessive bulbospinal neuronopathy. A clinicopathological study. Brain.

[3] Katsuno M., Tanaka F., Adachi H., Banno H., Suzuki K., Watanabe H., Sobue G. (2012). Pathogenesis and therapy of spinal and bulbar muscular atrophy (SBMA). Prog. Neurobiol..

[4] La Spada A.R., Wilson E.M., Lubahn D.B., Harding A.E., Fischbeck K.H. (1991). Androgen receptor gene mutations in X-linked spinal and bulbar muscular atrophy. Nature.

[5] Fischbeck K.H. (2012). Developing treatment for spinal and bulbar muscular atrophy. Prog. Neurobiol..

[6] Chua J.P., Lieberman A.P. (2013). Pathogenic mechanisms and therapeutic strategies in spinobulbar muscular atrophy. CNS Neurol. Disord. Drug Targets.

[7] Chua J.P., Reddy S.L., Merry D.E., Adachi H., Katsuno M., Sobue G., Robins D.M., Lieberman A.P. (2014). Transcriptional activation of TFEB/ZKSCAN3 target genes underlies enhanced autophagy in spinobulbar muscular atrophy. Hum. Mol. Genet..

[8] Yu Z., Wang A.M., Adachi H., Katsuno M., Sobue G., Yue Z., Robins D.M., Lieberman A.P. (2011). Macroautophagy is regulated by the UPR-mediator CHOP and accentuates the phenotype of SBMA mice. PLoS Genet..

[9] Jordan C.L., Lieberman A.P. (2008). Spinal and bulbar muscular atrophy: a motoneuron or muscle disease. Curr. Opin. Pharmacol..

[10] Cortes C.J., Ling S.C., Guo L.T., Hung G., Tsunemi T., Ly L., Tokunaga S., Lopez E., Sopher B.L., Bennett C.F. (2014). Muscle expression of mutant androgen receptor accounts for systemic and motor neuron disease phenotypes in spinal and bulbar muscular atrophy. Neuron.

[11] Lieberman A.P., Yu Z., Murray S., Peralta R., Low A., Guo S., Yu X.X., Cortes C.J., Bennett C.F., Monia B.P. (2014). Peripheral androgen receptor gene suppression rescues disease in mouse models of spinal and bulbar muscular atrophy. Cell Rep..

[12] Suzuki K., Katsuno M., Banno H., Takeuchi Y., Atsuta N., Ito M., Watanabe H., Yamashita F., Hori N., Nakamura T. (2008). CAG repeat size correlates to electrophysiological motor and sensory phenotypes in SBMA. Brain.

[13] Adachi H., Katsuno M., Minamiyama M., Waza M., Sang C., Nakagomi Y., Kobayashi Y., Tanaka F., Doyu M., Inukai A. (2005). Widespread nuclear and cytoplasmic accumulation of mutant androgen receptor in SBMA patients. Brain.

[14] Walcott J.L., Merry D.E. (2002). Ligand promotes intranuclear inclusions in a novel cell model of spinal and bulbar muscular atrophy. J. Biol. Chem..

[15] Chevalier-Larsen E.S., O'Brien C.J., Wang H., Jenkins S.C., Holder L., Lieberman A.P., Merry D.E. (2004). Castration restores function and neurofilament alterations of aged symptomatic males in a transgenic mouse model of spinal and bulbar muscular atrophy. J. Neurosci..

[16] Chevalier-Larsen E.S., Merry D.E. (2012). Testosterone treatment fails to accelerate disease in a transgenic mouse model of spinal and bulbar muscular atrophy. Dis. Mod. Mech..

[17] Poletti A. (2004). The polyglutamine tract of androgen receptor: from functions to dysfunctions in motor neurons. Front. Neuroendocrinol..

[18] Katsuno M., Adachi H., Kume A., Li M., Nakagomi Y., Niwa H., Sang C., Kobayashi Y., Doyu M., Sobue G. (2002). Testosterone reduction prevents phenotypic expression in a transgenic mouse model of spinal and bulbar muscular atrophy. Neuron.

[19] Schmidt B.J., Greenberg C.R., Allingham–Hawkins D.J., Spriggs E.L. (2002). Expression of X-linked bulbospinal muscular atrophy (Kennedy disease) in two homozygous women. Neurology.

[20] Katsuno M., Adachi H., Inukai A., Sobue G. (2003). Transgenic mouse models of spinal and bulbar muscular atrophy (SBMA). Cytogenet. Genome Res..

[21] Yu Z., Dadgar N., Albertelli M., Scheller A., Albin R.L., Robins D.M., Lieberman A.P. (2006). Abnormalities of germ cell maturation and sertoli cell cytoskeleton in androgen receptor 113 CAG knock-in mice reveal toxic effects of the mutant protein. Am. J. Pathol..

[22] Katsuno M., Banno H., Suzuki K., Takeuchi Y., Kawashima M., Yabe I., Sasaki H., Aoki M., Morita M., Nakano I. (2010). Efficacy and safety of leuprorelin in patients with spinal and bulbar muscular atrophy (JASMITT study): a multicentre, randomised, double-blind, placebo-controlled trial. Lancet Neurol..

[23] Adachi H., Waza M., Katsuno M., Tanaka F., Doyu M., Sobue G. (2007). Pathogenesis and molecular targeted therapy of spinal and bulbar muscular atrophy. Neuropathol. Appl. Neurobiol..

[24] Pozzi P., Bendotti C., Simeoni S., Piccioni F., Guerini V., Marron T.U., Martini L., Poletti A. (2003). Androgen 5-alpha-reductase type 2 is highly expressed and active in rat spinal cord motor neurones. J. Neuroendocrinol..

[25] Fernandez-Rhodes L.E., Kokkinis A.D., White M.J., Watts C.A., Auh S., Jeffries N.O., Shrader J.A., Lehky T.J., Li L., Ryder J.E. (2011). Efficacy and safety of dutasteride in patients with spinal and bulbar muscular atrophy: a randomised placebo-controlled trial. Lancet Neurol..

[26] Renier K.J., Troxell-Smith S.M., Johansen J.A., Katsuno M., Adachi H., Sobue G., Chua J.P., Sun Kim H., Lieberman A.P., Breedlove S.M. (2014). Anti-androgen flutamide protects male mice from androgen-dependent toxicity in three models of spinal bulbar muscular atrophy. Endocrinology.

[27] Orr C.R., Montie H.L., Liu Y., Bolzoni E., Jenkins S.C., Wilson E.M., Joseph J.D., McDonnell D.P., Merry D.E. (2010). An interdomain interaction of the androgen receptor is required for its aggregation and toxicity in spinal and bulbar muscular atrophy. J. Biol. Chem..

[28] Montie H.L., Cho M.S., Holder L., Liu Y., Tsvetkov A.S., Finkbeiner S., Merry D.E. (2009). Cytoplasmic retention of polyglutamine-expanded androgen receptor ameliorates disease via autophagy in a mouse model of spinal and bulbar muscular atrophy. Hum. Mol. Genet..

[29] Montie H.L., Pestell R.G., Merry D.E. (2011). SIRT1 modulates aggregation and toxicity through deacetylation of the androgen receptor in cell models of SBMA. J. Neurosci..

[30] Lieberman A.P., Harmison G., Strand A.D., Olson J.M., Fischbeck K.H. (2002). Altered transcriptional regulation in cells expressing the expanded polyglutamine androgen receptor. Hum. Mol. Genet..

[31] Lieberman A.P., Robitaille Y., Trojanowski J.Q., Dickson D.W., Fischbeck K.H. (1998). Polyglutamine-containing aggregates in neuronal intranuclear inclusion disease. Lancet.

[32] Thomas M., Harrell J.M., Morishima Y., Peng H.M., Pratt W.B., Lieberman A.P. (2006). Pharmacologic and genetic inhibition of hsp90-dependent trafficking reduces aggregation and promotes degradation of the expanded glutamine androgen receptor without stress protein induction. Hum. Mol. Genet..

[33] Thomas M., Yu Z., Dadgar N., Varambally S., Yu J., Chinnaiyan A.M., Lieberman A.P. (2005). The unfolded protein response modulates toxicity of the expanded glutamine androgen receptor. J. Biol. Chem..

[34] Thomas M., Dadgar N., Aphale A., Harrell J.M., Kunkel R., Pratt W.B., Lieberman A.P. (2004). Androgen receptor acetylation site mutations cause trafficking defects, misfolding, and aggregation similar to expanded glutamine tracts. J. Biol. Chem..

[35] Wang A.M., Miyata Y., Klinedinst S., Peng H.M., Chua J.P., Komiyama T., Li X., Morishima Y., Merry D.E., Pratt W.B. (2013). Activation of Hsp70 reduces neurotoxicity by promoting polyglutamine protein degradation. Nat. Chem. Biol..

[36] Morishima Y., Wang A.M., Yu Z., Pratt W.B., Osawa Y., Lieberman A.P. (2008). CHIP deletion reveals functional redundancy of E3 ligases in promoting degradation of both signaling proteins and expanded glutamine proteins. Hum. Mol. Genet..

[37] Rusmini P., Crippa V., Giorgetti E., Boncoraglio A., Cristofani R., Carra S., Poletti A. (2013). Clearance of the mutant androgen receptor in motoneuronal models of spinal and bulbar muscular atrophy. Neurobiol. Aging.

[38] Rusmini P., Sau D., Crippa V., Palazzolo I., Simonini F., Onesto E., Martini L., Poletti A. (2007). Aggregation and proteasome: the case of elongated polyglutamine aggregation in spinal and bulbar muscular atrophy. Neurobiol. Aging.

[39] Simeoni S., Mancini M.A., Stenoien D.L., Marcelli M., Weigel N.L., Zanisi M., Martini L., Poletti A. (2000). Motoneuronal cell death is not correlated with aggregate formation of androgen receptors containing an elongated polyglutamine tract. Hum. Mol. Genet..

[40] Piccioni F., Pinton P., Simeoni S., Pozzi P., Fascio U., Vismara G., Martini L., Rizzuto R., Poletti A. (2002). Androgen receptor with elongated polyglutamine tract forms aggregates that alter axonal trafficking and mitochondrial distribution in motor neuronal processes. FASEB J..

[41] Rusmini P., Bolzoni E., Crippa V., Onesto E., Sau D., Galbiati M., Piccolella M., Poletti A. (2010). Proteasomal and autophagic degradative activities in spinal and bulbar muscular atrophy. Neurobiol. Dis..

[42] Castillo K., Nassif M., Valenzuela V., Rojas F., Matus S., Mercado G., Court F.A., van Zundert B., Hetz C. (2013). Trehalose delays the progression of amyotrophic lateral sclerosis by enhancing autophagy in motoneurons. Autophagy.

[43] Tanaka M., Machida Y., Niu S., Ikeda T., Jana N.R., Doi H., Kurosawa M., Nekooki M., Nukina N. (2004). Trehalose alleviates polyglutamine-mediated pathology in a mouse model of Huntington disease. Nat. Med..

[44] Sarkar S., Davies J.E., Huang Z., Tunnacliffe A., Rubinsztein D.C. (2007). Trehalose, a novel mTOR-independent autophagy enhancer, accelerates the clearance of mutant huntingtin and alpha-synuclein. J. Biol. Chem..

[45] Davies J.E., Sarkar S., Rubinsztein D.C. (2006). Trehalose reduces aggregate formation and delays pathology in a transgenic mouse model of oculopharyngeal muscular dystrophy. Hum. Mol. Genet..

[46] Aguib Y., Heiseke A., Gilch S., Riemer C., Baier M., Schatzl H.M., Ertmer A. (2009). Autophagy induction by trehalose counteracts cellular prion infection. Autophagy.

[47] Lan D.M., Liu F.T., Zhao J., Chen Y., Wu J.J., Ding Z.T., Yue Z.Y., Ren H.M., Jiang Y.P., Wang J. (2012). Effect of trehalose on PC12 cells overexpressing wild-type or A53 T mutant alpha-synuclein. Neurochem. Res..

[48] Zhang X., Chen S., Song L., Tang Y., Shen Y., Jia L., Le W. (2014). MTOR-independent, autophagic enhancer trehalose prolongs motor neuron survival and ameliorates the autophagic flux defect in a mouse model of amyotrophic lateral sclerosis. Autophagy.

[49] Montie H.L., Merry D.E. (2009). Autophagy and access: understanding the role of androgen receptor subcellular localization in SBMA. Autophagy.

[50] Yu Z., Wang A.M., Robins D.M., Lieberman A.P. (2009). Altered RNA splicing contributes to skeletal muscle pathology in Kennedy disease knock-in mice. Dis. Mod. Mech..

[51] Adachi H., Waza M., Tokui K., Katsuno M., Minamiyama M., Tanaka F., Doyu M., Sobue G. (2007). CHIP overexpression reduces mutant androgen receptor protein and ameliorates phenotypes of the spinal and bulbar muscular atrophy transgenic mouse model. J. Neurosci..

[52] Rusmini P., Simonini F., Crippa V., Bolzoni E., Onesto E., Cagnin M., Sau D., Ferri N., Poletti A. (2011). 17-AAG increases autophagic removal of mutant androgen receptor in spinal and bulbar muscular atrophy. Neurobiol. Dis..

[53] Carra S., Rusmini P., Crippa V., Giorgetti E., Boncoraglio A., Cristofani R., Naujock M., Meister M., Minoia M., Kampinga H.H. (2013). Different anti-aggregation and pro-degradative functions of the members of the mammalian sHSP family in neurological disorders. Philos. Trans. R. Soc. Lond. B Biol. Sci..

[54] Crippa V., Sau D., Rusmini P., Boncoraglio A., Onesto E., Bolzoni E., Galbiati M., Fontana E., Marino M., Carra S. (2010). The small heat shock protein B8 (HspB8) promotes autophagic removal of misfolded proteins involved in amyotrophic lateral sclerosis (ALS). Hum. Mol. Genet..

[55] Crippa V., Carra S., Rusmini P., Sau D., Bolzoni E., Bendotti C., De Biasi S., Poletti A. (2010). A role of small heat shock protein B8 (HspB8) in the autophagic removal of misfolded proteins responsible for neurodegenerative diseases. Autophagy.

[56] Liu R., Barkhordarian H., Emadi S., Park C.B., Sierks M.R. (2005). Trehalose differentially inhibits aggregation and neurotoxicity of beta-amyloid 40 and 42. Neurobiol. Dis..

[57] Wang X., Fan H., Ying Z., Li B., Wang H., Wang G. (2010). Degradation of TDP-43 and its pathogenic form by autophagy and the ubiquitin-proteasome system. Neurosci. Lett..

[58] Cashman N.R., Durham H.D., Blusztajn J.K., Oda K., Tabira T., Shaw I.T., Dahrouge S., Antel J.P. (1992). Neuroblastoma x spinal cord (NSC) hybrid cell lines resemble developing motor neurons. Devel. Dyn..

[59] Durham H.D., Dahrouge S., Cashman N.R. (1992). Evaluation of the spinal cord neuron X neuroblastoma hybrid cell line NSC-34 as a model for neurotoxicity testing. *Neurotoxicol*ogy.

[60] Marron T.U., Guerini V., Rusmini P., Sau D., Brevini T.A.L., Martini L., Poletti A. (2005). Androgen-induced neurite outgrowth is mediated by neuritin in motor neurones. J. Neurochem..

[61] Sau D., De Biasi S., Vitellaro-Zuccarello L., Riso P., Guarnieri S., Porrini M., Simeoni S., Crippa V., Onesto E., Palazzolo I. (2007). Mutation of SOD1 in ALS: a gain of a loss of function. Hum. Mol. Genet..

[62] Onesto E., Rusmini P., Crippa V., Ferri N., Zito A., Galbiati M., Poletti A. (2011). Muscle cells and motoneurons differentially remove mutant SOD1 causing familial amyotrophic lateral sclerosis. J. Neurochem..

[63] Poletti A., Rampoldi A., Piccioni F., Volpi S., Simeoni S., Zanisi M., Martini L. (2001). 5Alpha-reductase type 2 and androgen receptor expression in gonadotropin releasing hormone GT1–1 cells. J. Neuroendocrinol..

